# New prognosis inflammatory and nutritional indexes: comparison with the Prognostic Inflammatory and Nutritional Index as reference index

**DOI:** 10.1186/cc9918

**Published:** 2011-03-11

**Authors:** F Ziegler, L Codevelle, E Houivet, J Benichou, A Lavoinne, P Dechelotte

**Affiliations:** 1 Université et CHU, Rouen, France

## Introduction

The Prognostic Inflammatory and Nutritional Index (PINI) was previously developed to improve the diagnosis and monitoring of patients with inflammation and/or malnutrition in terms of morbidity and mortality risk, especially in ICU patients [1]. The formula includes the determination of four serum protein concentrations: PINI = (C-reactive protein (CRP) (mg/l) × orosomucoid (OROSO) (g/l))/(albumin (ALB) (g/l) × transthyretin (TTR) (g/l)). Since CRP may be considered now as the gold standard for assessing and monitoring inflammatory states in clinical practice, OROSO is generally unavailable for PINI calculation. Elsewhere, the strong and rapid changes in CRP levels (0 to 600 mg/l) in acute inflammation may lead to an overestimation of the risk of morbidity and mortality suggested by the PINI. The aim of this study was to evaluate alternative biological formulas by removing OROSO from the PINI and replacing CRP value by its logarithm (Log), in order to reduce the mathematical weighting of this biomarker.

## Methods

Blood samples of 106 patients hospitalized in intensive care, gastrointestinal surgery, vascular and thoracic surgery, pneumology, gastroenterology or internal medicine units were drawn to measure serum concentrations of ALB, TTR, CRP and OROSO. Proteins were determined using an immunonephelometry method (BN2; Siemens, Germany). The correlations between six new formulas and the PINI were studied - that is, CRP/ALB × TTR, Log(CRP)/ALB × TTR, CRP/TTR, Log(CRP)/TTR, CRP/ALB and Log(CRP)/ALB - using the Spearman rank test.

## Results

The relations obtained between the PINI and the experimental formulas were linear (*y *= *ax *+ *b*) with formulas without Log and nonlinear when a Log was used (*y *= *ax*^2 ^+ *bx *+ *c *or *y *= log(*x*) + *b*). All six formulas were correlated with the PINI (0.78 <*R *< 0.94, *P *< 0.0001). CRP/ALB × TTR, Log(CRP)/ALB and CRP/TTR showed the highest correlations, with *R *= 0.94, 0.91 and 0.90, respectively. The less elevated correlation was observed using CRP/ALB (*R *= 0.78).

## Conclusions

Among the six new formulas compared with the PINI, that omitting only OROSO provided the best performance. The control of CRP weighting obtained with Log(CRP) in the formula Log(CRP)/ALB appears promising in current clinical practice, since it involves the most often used serum proteins to assess inflammatory and nutritional status.
